# The Magazines

**Published:** 1873-01

**Authors:** 


THE MAGAZINES. *The Atlantic Monthly* commences the year with a number filled with interesting and instructive articles. Jas. Parton contributes a valuable paper on Washington and his Cabinet, and O. W. Holmes, has a poem entitled "After the Fire."

The February number contains papers from the pens of J. V. Blake, Robt. Dale Owen, Jas. Parton, and others. John G. Whittier contributes a poem entitled "A Mystery," which is full of pleasant and instructive thoughts.

*To-Day* is the title of a Magazine edited by Dio Lewis, and published by Maclean Stoddart & Co. The Magazine is published weekly and is finely illustrated. The Christmas number is exceedingly interesting, and is filled with the usual variety of matter found in periodical publications. The contents are of a varied nature, some amusing and witty, others grave and instructive and all combined, furnish a weekly magazine which can not help being appreciated by all into whose hands it may come.

